# A systematic review of factors associated with student use of campus food pantries: implications for addressing barriers and facilitating use

**DOI:** 10.1186/s12889-023-17583-7

**Published:** 2024-01-05

**Authors:** Oisemujaime Victoria Idehai, Pindar Mbaya, Tammy Chung, Trishnee Bhurosy

**Affiliations:** 1 Department of Population Health, School of Health Professions and Human Services, Hofstra University, Hempstead, NY 11549 USA; 2 Center for Population Behavioral Health, Rutgers the State University of New Jersey, New Brunswick, NJ 08901 USA; 3 Department of Nutrition and Food Sciences, College of Agriculture and Life Sciences, University of Vermont, Burlington, VT 05405 USA

**Keywords:** Food insecurity, College students, Campus food pantries, Barriers, Predictors, Facilitators

## Abstract

**Background:**

While campus food pantries have been important safety net programs for alleviating food insecurity among college students, factors related to accessing these vital resources have not been fully researched and summarized. This study systematically synthesized peer-reviewed literature on the predictors, barriers to, and facilitators of using campus food pantries among college students.

**Methods:**

A search was conducted on PubMed, CINAHL Complete, PsychInfo, PsycARTICLES, and ScienceDirect in April 2023. Included studies needed to be peer-reviewed, written in English, and focused on college or university students. Three authors independently screened all articles retrieved from the five databases based on titles, titles and abstracts, and a full article review. The Study Quality Assessment Tool from the National Heart, Lung, and Blood Institute was used to assess the risk of bias in the included cross-sectional studies. The risk of bias and quality of mixed methods or qualitative studies were assessed as well.

**Results:**

Eight studies were included in the systematic review. Students likely to use a college food pantry were food-insecure, who most often identified as Asian, Hispanic/Latino, Filipino or Pacific Islander; were first-generation to college; international students; sophomores and juniors; had student loans; were living off-campus; and were without stable housing. Stigma was the most frequently mentioned barrier to using a food pantry. Participants mentioned facilitators such as convenient location and hours of operation, access to fresh produce and nutritious and safe foods, availability of a variety of foods, friendly and helpful service, social support, and awareness of a pantry through fellow students and other members of the university such as staff and faculty.

**Conclusions:**

Continued research must address students' systemic barriers to accessing food pantries. Campus food pantry leaders, university administrators, and policymakers need to work together to create cost-effective and sustainable solutions that will alleviate the stigma and burden of food-insecure students and provide them with safe, nutritious, and culturally acceptable foods.

## Introduction

Over the past decade, college students have been reported as an emerging population at risk for food insecurity [1–3]. Food insecurity, defined as the limited or uncertain availability of nutritionally adequate and safe foods by the United States (US) Department of Agriculture [4], affects nearly fifty million people in the US, making it one of the nation’s leading health issues [5]. Food insecurity affects between 10 to 75% of college students in the US, putting them at risk for depression, poor academic performance, low quality of life, and social isolation [2, 3]. During and after the COVID-19 pandemic, college students reported experiencing more academic issues due to food insecurity, in addition to higher housing insecurity and less access to healthcare [6, 7].

Most federal food assistance programs do not prioritize food-insecure college students unless stringent exceptions are met [8, 9]. Hence, campus food pantries are critical safety net programs designed to alleviate food deprivation and hunger among college students [10]. A large cross-sectional study of 1,855 students reported that a greater number of campus food pantry visits was associated with improved perceived health, decreased depressive symptoms, and better sleep sufficiency [11]. Despite the existence of food pantries on college campuses, students might still struggle to access food due to several reasons. Some of these reasons included social stigma and embarrassment, insufficient information on how the program works, lack of information regarding the eligibility criteria, lack of measures to protect confidentiality, and inconvenient hours of operation [12].

While many recent studies have reported on the high rates and increase in food insecurity prevalence [13, 14], factors, including challenges and facilitators, related to food pantry usage among college students have not been fully researched and summarized. Based on The Stigma and Food Inequity Framework, there are structural and individual levels of stigma that are mediated by different factors, including access to resources, food environment, and psychosocial and behavioral processes [15]. We chose the Stigma and Food Inequity Conceptual Framework for three reasons. There are only a few comprehensive frameworks that have been developed to better understand factors related to food insecurity and stigma. Second, the Stigma and Food Inequity Framework was chosen because of its usefulness in categorizing both downstream and upstream factors related to food insecurity and stigma [15]. Lastly, it was recently developed based on findings from prior conceptual and empirical stigma research in public health [15].

Hence, this study will explore individual factors (e.g., demographic and student characteristics), psychosocial and behavioral factors (e.g., perception of the pantry), and social and structural determinants (e.g., college infrastructure and access to resources) that are related to food pantry usage among college students. This study has two objectives: (1) systematically review and summarize peer-reviewed literature on the predictors, barriers to, and facilitators of using campus food pantries among college students and (2) identify opportunities for research, practice, and policy to improve usage of food pantries among college students. To the best of the authors’ knowledge, no other systematic reviews have been conducted related to the current study’s aims.

## Methods

### Search strategy

The protocol for this review has been registered on the International Prospective Register for Systematic Reviews (PROSPERO) (Registration ID: 418831). In accordance with the Preferred Reporting Items for Systematic Reviews and Meta-Analysis (PRISMA) guidelines, the authors searched for studies that examined the barriers to, facilitators, and predictors of utilizing campus food pantries among college students. The corresponding author (TB) met with a research librarian to refine the search syntax and together with VOI and PM, generated a list of search terms. A search was conducted on PubMed, CINAHL Complete, PsychInfo, PsycARTICLES, and ScienceDirect using search terms such as “food pantry” or “food pantries” OR “food bank” OR “food banks” AND “college” OR “colleges” OR “university” OR “universities” OR “student” OR “students” OR “undergraduate” OR “undergraduates” OR “graduate” OR “graduates”. This review did not involve human subjects, thus approval from the institutional review board was therefore not required.

### Inclusion/exclusion criteria

Inclusion criteria specified studies that assessed any barriers, facilitators, and possible predictors or determinants of using campus food pantries from the inception date of each database to April 14, 2023, written in English, and with the priority population being college or university students or any other groups of students at a higher educational institution. Studies having either a descriptive (e.g., surveys and case studies) or observational (e.g., cohort studies) research design were considered for inclusion. This search included studies that employed qualitative and/or mixed methods. Studies that focused on faculty and staff within a college or university setting were excluded. Other exclusion criteria included pre-prints, books, narrative reviews, systematic reviews, meta-analyses, research abstracts, conference proceedings, and studies whose methodologies were not clear.

### Study selection and data extraction

**Fig. 1:**
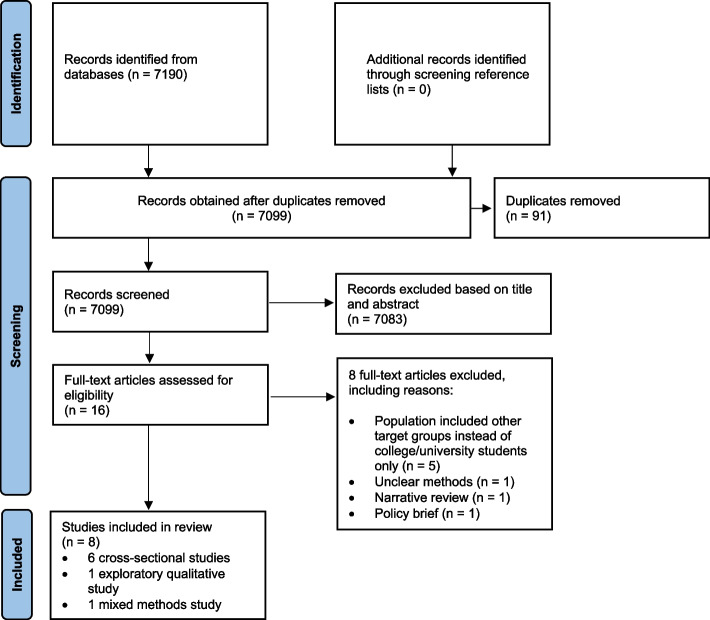
PRISMA figure showing selection, screening, and reviewing of studies

The PRISMA flow chart (Fig. 1) shows the steps in the study selection process. Using Zotero, study authors (VOI, PM, and TB) independently screened all articles retrieved from the five databases based on titles, titles and abstracts, and a full article review (Fig. 1). The three authors then met twice to discuss and mutually resolve any discrepancies using the pre-established inclusion and exclusion criteria.

### Study quality and risk of bias in studies

This study assessed the risk of bias in studies using the Study Quality Assessment Tools for cross-sectional studies by the National Heart, Lung, and Blood Institute [16]. Examples of questions used to assess study quality included “Was the research question or objective in this paper clearly stated?; Was the study population clearly specified and defined?; Was the participation rate of eligible persons at least 50%?; Were all the subjects selected or recruited from the same or similar populations?; Were inclusion and exclusion criteria for being in the study prespecified and applied uniformly to all participants?; Was a sample size justification, power description, or variance and effect estimates provided?; Were key potential confounding variables measured and adjusted statistically for their impact on the relationship between exposure (s) and outcome(s)?” To assess the risk of bias and quality of the qualitative and mixed methods study, we used the following guidelines by Long and Godfrey [17] that have been used in another systematic review [18]: “ Was a research question clearly stipulated; were key characteristics of participants provided; was the qualitative approach appropriate to answer the main research question; were the data collection methods sufficiently presented; was there sufficient breadth to the findings elicited from participants; were the findings discussed within the context of other studies and did the authors identify any potential biases?” Zero was assigned to items that were missing or unclear while one was given for criteria that were met by the study [18].

## Results

### Study selection

A search from the databases yielded 7,190 articles (Fig. 1). No additional articles were found after the authors carefully reviewed the reference lists of all included articles. We screened 7,099 articles after duplicates were removed. We excluded 7,083 articles based on their titles and abstracts. Out of 7,083 articles, 5,032 focused on health issues such as the Zika virus, zoonotic diseases, mental health and psychological distress, breastfeeding, mastitis, water insecurity, men’s health, food addictions, child hunger, organizational malpractices, and migrant health among others; 1004 were abstracts, conference proceedings or position statements, 765 articles were either systematic or narrative issues that did not fit the inclusion criteria; and 282 articles did not specifically focus on the factors related to campus food pantry usage among university or college students. We then assessed 16 full-text articles for their eligibility. Eight articles were excluded based on the following reasons. Five articles focused on an adult population other than college or university students or students within a higher educational institution and three articles were either a narrative review, a policy brief, or had unclear methods. A final list of eight articles was included in this systematic review.

### Study and participant characteristics

Six out of eight studies in this current systematic review employed a cross-sectional research design [12, 19–23]. One study used an exploratory qualitative design [24] and another used a mixed methods research design [25] (see Table 1). The studies were conducted in Texas, Florida, California, Kentucky, Illinois, and New Jersey [12, 19–25]. Five studies recruited convenience samples through email listservs, flyers, Basic Needs Centers, or campus food pantries [12, 19, 20, 24, 25]. The remaining three studies used a random sample of enrolled students [21–23]. One cross-sectional study was based on only campus food pantry users [25] and another recruited participants from Campus Basic Needs Centers [20]. The range of campus food pantry users varied from 2.3% to 10.5% in three other studies [19, 21, 22]. Most studies recruited participants who were predominantly undergraduate (range: 65.6 – 80%), female (range: 54.9 – 93.9%), and living off-campus (range: 70 – 100%). Almost half of the studies recruited a diverse sample of racial and ethnic groups, including Asian (range: 6 – 37%), Black (range: 4 – 12.9%), Latino (range: 22.5 – 44%), or mixed race/ethnicity (range: 10.4 – 17.1%) [19, 20, 24, 25].

**Table 1:** Characteristics and key findings of included studies

Authors, year	Region	Research Design, Sample Size, and Recruitment	Participant Characteristics	Key Findings on Predictors	Key Findings on Facilitators	Key Findings on Barriers
Brito-Silva et al., 2022 [19]	Denton, Texas	• Cross-sectional (*N* = 575) • Convenience sampling through email invitations	• Participants were mostly female (93.9%), under 25 (69.1%), full-time students (84.7%), and living off-campus (72.4%) • 40.1% identified as White, 12.9% as African American, 22.5% as Hispanic/Latino, 11.0% as Asian, and 10.4% as multiple races or ethnicities • 52 (9.0%) used a campus food pantry (CFP)	• Asian students were more likely to use a CFP than White students [Adjusted Odds Ratio (OR) = 3.4; 95% confidence intervals (CI): 1.28–9.12] • First-year students (OR = 0.08; 95% CI: 0.013–0.470), sophomores (OR = 0.13; 95% CI: 0.03–0.65), and juniors (OR = 0.27; 95% CI: 0.07–0.99) were more likely to use a CFP • Students in the low food security category (OR = 3.4; 95% CI: 1.49–7.82) and very low food security category (OR = 5.02; 95% CI: 2.22–11.36) were more likely to use a CFP	• Participants suggested solutions to address barriers such as flexibility of access, clearing out expired food, providing fresh produce, increased marketing and advertising, and investment to destigmatize food insecurity	• 248 (47.8%) students were unaware of on-campus food pantries’ services • Doctoral students were more likely to report barriers than first-year and junior students • 130 students (26.9%) perceived barriers to accessing campus food pantries, including being a full-time student, transportation issues, embarrassing questions about accessing the pantry, the feeling of not deserving or needing the pantry, lack of information about the pantry’s existence, operation, eligibility, lack of time, food pantry location, poor food quality, reduced hours of operation, and social stigma of being food insecure
El Zein et al., 2018 [12]	Gainesville, Florida	• Cross-sectional (*N* = 899) • Convenience sampling through email invitations	• Participants were mostly female (74.3%), White (77.6%), non-Hispanic/Latino (82.1%), single (85.6%), and undergraduate (65.6%) • Almost half did a part-time or full-time job (49.7%) • About 35% had a student loan and 22.9% were Pell Grant recipients	• Pell Grant recipients (OR = 1.87; 95% CI = 1.01–3.48), those receiving student loans (OR = 2.31, 95% CI = 1.39–3.82), international students (OR = 7.16; 95% CI = 3.13–16.35) and food insecure students (OR = 8.85; 95% CI = 5.13–15.26) were more likely to use the CFP than their counterparts	• No findings reported	• 68 (12.7%) students experienced barriers to using the CFP • Out of the 68 students, 51.5% who were food insecure reported barriers to using the CFP • Main barriers reported by participants were social stigma and embarrassment (36.8%), insufficient information on how the program works and what determines eligibility (33.8%), the feeling that the food was not for them (17.6%), and inconvenient hours of operation (11.8%)
El Zein et al., 2022 [24]	Gainesville, Florida	• Exploratory descriptive (*N* = 41) • Participants recruited at the CFP, through flyers, and e-mail listservs	• Each participant completed a one-on-one, in-person, semi-structured interview • Participants were mainly women (70.7%) with an average age of 23.7 ± 5.9 years, White (46.3%), Asian (26.8%), multi-racial (17.1%), and Black (9.8%) • Most participants were undergraduates (70.8%) and 63.4% were employed (part-time/full-time) • 26.8% were international students • 58.5% had financial aid and 34.1% were Pell Grant recipients	• No findings reported	• Participants suggested three solutions to minimize barriers experienced while utilizing the CFP: awareness through positive marketing messages, improving accessibility of fresh produce and protein options, and improving access through satellite locations and online ordering systems	• Participants reported barriers such as stigma and shame, perceived insufficient need, unsuitable food, lack of knowledge, and limited food access
Esaryk et al., 2021 [20]	California	• Cross-sectional (*N* = 1,513) • Participants were recruited through the listservs of the campus Basic Needs Centers	• All participants had used a CFP at least once • Participants were mostly female (78%), living off-campus (70%), first-generation students (59%), received need-based financial aid (79%), and had a median age of 21 • 38% of participants were Latino, 37% were Asian, 14% were White, 5% were Filipino or Pacific Islander, 4% were Black, and 1% were American Native	• Students who had received need-based financial need, were male, older, or first-generation were more likely to have more frequent CFP visits • Filipino/Pacific Islanders visited the CFP more times than White students • Off-campus students and those without stable housing were more likely to visit the CFP than on-campus students • Incidence ratios were not provided for these above findings	• Participants reported facilitators such as awareness through friends/fellow students (69%), food pantry/basic needs staff (28%), social media (31%), student peer advisor (17%), print or other media (12%), faculty (11%), workshop or presentation attended (11%), and referral from another campus service (5%)	• No findings reported
Manboard et al., 2021 [25]	Texas	• Cross-sectional (*N* = 18) • Participants visiting the CFP were recruited	• Most participants were females (83%) and all participants lived off-campus • 44% identified as Latino or Hispanic, 28% as White, 11% as Black, 6% as Asian, and 12% as mixed races and ethnicities	• No findings reported	• Participants reported social support, social networks (friends, families, roommates, campus, and local community groups), relief funds during the pandemic, and mental health counseling as facilitators	• Participants reported barriers, including a lack of physical access and a lack of systematic assistance within their educational institution
McArthur et al., 2020 [21]	Kentucky	• Cross-sectional (*N* = 896; 14.9% response rate) • Participants were recruited from a computer-generated randomized sample	• About 70% of the participants were female, about 90% were full-time students, 80% were undergraduates, about 80% were non-Hispanic whites, 55% were employed, 25% participated in an on-campus meal plan, and 25% lived on-campus • 94 students (10.5%) had used the CFP at least once	• Among pantry users, about two-thirds were female, had a mean age of 21.8, and two-thirds were non-Hispanic whites • 50% of the pantry users were employed, 20% had an on-campus meal plan, and 4% participated in a state or federal food assistance program	• CFP users strongly agreed or agreed on statements such as foods were safe to eat (85.1%), familiar foods were available (83.0%), organized inventory (81.9%), friendly service (79.8%), helpful service (77.6%), spacious/roomy (77.6%), convenient location (72.5%), open at convenient times, (66.0%), nutritious/healthful foods were available (61.7%), and visually appealing (62.7%)	• 30.1% of participants felt others needed the CFP more than they did, 20.7% felt embarrassed asking for help, and 12.6% did not know how to ask for help • Other barriers reported were schedule conflicts with hours of pantry operation, lack of cooking equipment, and not finding foods they liked • About 27% of food pantry users strongly agreed or agreed that culturally diverse foods were available
Mitchell et al., 2022 [22]	Illinois	• Cross-sectional (*N* = 888; 17.8% response rate) • A random sample of participants was invited to participate	• The sample was predominantly undergraduate (79.7%), food secure (78.2%), US-born (79.8%), female (54.9%), and 22 years or older (58.8%) • 180 students (20.2%) were aware of the pantry and 20 (2.3%) used it • 23% of food-insecure students were aware of the CFP and 60% of food-insecure students used the CFP	• Food insecure students (*p* < 0.001), those facing episodic (*p* = 0.05) or chronic food insecurity (*p* < 0.001), Hispanic/Latinx students (*p* = 0.007), and those receiving federal financial support (*p* = 0.002) reported using the campus food pantry more than others	• On a scale of 1 to 5 with 5 being very satisfied, participants reported a mean of 4.5 on their satisfaction level with the variety of foods and number of healthy foods from the pantry, a mean of 4.5 on receiving help promptly, and a mean of 4.3 on convenient hours of the pantry	• 30% of food-insecure students reported not using the CFP because they were uncomfortable • Reasons for dissatisfaction were expired food or produce, foods nearing the expiration date, and short hours of operation
Weaver et al., 2022 [23]	New Jersey	• Cross-sectional (*N* = 1,374; 15% response rate) • All undergraduates enrolled at the university were invited to participate	• 375 participants were in a very low food security status • Participants with very low food security had a mean age of 21 and a mean GPA of 3.04 • 56.7% of students in the very low food security status were female; 66.3% were first-generation; and 81.7% were African American	• No findings reported	• Feeling grateful and appreciative, and perceiving the food pantry as a helpful place were positive emotions reported by participants if they had to use the CFP	• Participants with both high and low food security were concerned about stigma and the perception that other students needed the CFP more than them deterred them from using it

### Predictors of using campus food pantries

Based on the Stigma and Food Inequity Framework [15], this systematic review found sociodemographic and other characteristics related to food pantry use on campus among college or university students. Participants who were more likely to use a campus food pantry were food-insecure (either chronic or episodic) [12, 19, 22], those on student loans or receiving federal financial support [12, 20–22], Asian students [19], Hispanic/Latino students [22], Filipino or Pacific Islander [20], first-generation [20], undergraduates [19], international students [12], Pell Grant recipients [12], and those living off-campus [20] and without stable housing [20]. While a large cross-sectional study in California (survey recruitment conducted through Campus Basic Needs Centers listservs) reported that males were likely to use food pantries on campus [20], another study conducted in Kentucky (random sample of enrolled students) found that two-thirds of their pantry users were females [21].

### Facilitators of using campus food pantries

Facilitators of student access to food pantries within their educational institutions included flexibility in accessing the pantry through satellite locations and online ordering systems [19, 24]; access to fresh produce and protein options [24]; awareness through positive messages [24], fellow students, roommates, local community groups, student peer advisors, faculty and food pantry staff [20, 25]; referrals from another campus service, social media, print or other media; and workshops or presentations students attended [20]. Participants also mentioned facilitators such as accessing relief funds during crises such as a pandemic and receiving mental health counseling [20]. Other factors that helped campus food pantry users were the availability and variety of safe and familiar foods (e.g., spices, sauces, fresh produce, sandwiches), friendly and helpful service, spacious and convenient locations, convenient hours of operation, and access to nutritious and visually appealing foods [21, 22]. In addition, participants in one cross-sectional study reported positive emotions of gratitude and appreciation and perceived the campus food pantry as a helpful place [23].

### Barriers to using campus food pantries

Stigma was one common barrier reported by most studies [12, 19, 21–24]. Based on the Stigma and Food Inequity Framework, stigma manifestations among participants could be categorized into structural, stigma perceived by other people, and internalized or anticipated stigma [15]. Students reported structural stigma through messages promoted by their institutions such as having a competitive spirit “that leads to a false sense of not wanting to rely on anyone and try not to be seen as weaker in the fight” [24]. For instance, participants reported stigma perceived by other people through comments such as “possibly judgement from other students and/or having to justify the need is embarrassing” [19], and feeling afraid of being seen carrying pantry bags on campus and getting strange looks from other students [24]. Participants also reported internalized stigma through perceptions that going to the pantry was associated with stigma and they did not want to be seen as someone who needed help with basic needs such as food [12, 23, 24].

In addition to stigma, the review identified other psychosocial and structural barriers to campus food pantry use. Psychosocial barriers manifested through perceptions among participants that others needed the pantry more than them, that is, the student was taking away resources that others could use, or that the student was not “poor enough” [12, 21, 23]. Additional factors that hindered students from accessing food pantries on university campuses were lack of awareness and information about the pantry’s existence and operation, being asked embarrassing questions, the feeling of not needing the food pantry, and being a full-time or doctoral student [12, 19, 21, 22, 24, 25]. Some students did not have adequate cooking equipment to make use of available pantry items [21].

College infrastructure barriers to student use of the pantry included inadequate systemic assistance from the educational institution, transportation issues, time conflicts with operating hours of the pantry (reduced or short hours of pantry operation), inconvenient location of the food pantry, poor quality of food (e.g., expired food or food nearing the expiration date), and lack of culturally diverse foods [12, 19, 21, 22, 24, 25]. Other barriers that participants experienced were insufficient information on how food pantry programs worked, and unclear eligibility criteria [12, 19, 22, 23]. For example, some students reported that they thought they were not eligible because of their international status [19].

### Quality and risk of bias in included studies

Table 2 shows the rating for each criterion of all studies. All studies included their research aims or research questions (see Table 2). Most studies defined their inclusion and exclusion criteria [19, 20, 23–25]. Three studies scored 0 on not providing information about the inclusion and exclusion criteria they used to recruit participants [12, 21, 22]. One cross-sectional study [22] did not provide a sample size justification. Most studies scored 0 on their sampling methods. Only three studies recruited their participants from a random sample, with response rates ranging from 14.9% to 17.8% (Tables 1 and 2) [21–23]. Two studies relied on convenience samples [12, 19]. Brito-Silva and colleagues noted that their sample was drawn from a primarily female, diverse, state-funded university in Texas [19]. In addition, two studies did not provide a justification for how they achieved their final sample sizes [22, 24]. The two studies that employed a qualitative or mixed methods research design provided clear research questions or aims, described key characteristics of their sample, provided sufficient details on their data collection methods, discussed their findings within the context of other studies, and identified specific limitations (Table 2). Confounding variables were not adjusted in analyses for two studies [21, 22]. Participants were recruited at public state universities in all studies.

**Table 2:** Assessment of risk of bias of included studies in the systematic review

Authors (year)	Was the research question or objective clearly stated and appropriate?	Was the study population clearly specified and defined?	Was random sampling used?	Were inclusion and exclusion criteria for being in the study prespecified and applied uniformly to all participants?	Was a sample size justification or power description, provided?	Were the measures clearly defined and implemented consistently across all study participants?	Were key potential confounding variables measured and adjusted in the analyses?
Brito-Silva et al., 2022 [19]	1	1	0	1	1	1	1
El-Zein et al., 2018 [12]	1	1	0	0	1	1	1
El-Zein et al., 2022 [24]	1	1	0	1	0	1	NA
Esaryk et al., 2021 [20]	1	1	0	1	1	1	1
Manboard et al., 2021 [25]	1	1	0	1	0	1	NA
McArthur et al., 2020 [21]	1	1	1	0	1	1	0
Mitchell et al., 2022 [22]	1	1	1	0	0	1	0
Weaver et al., 2021 [23]	1	1	1	1	1	1	NA

^*^0 Cannot be determined or not reported, *1* Reported, *NA* Not applicable

## Discussion

To the best of the authors’ knowledge, this is the first systematic review to examine the predictors and facilitators of and barriers to using campus food pantries among college students. This review consolidates current knowledge about factors related to the usage of campus food pantries while identifying specific sub-groups of college students who warrant the most attention in terms of improving access to these vital campus community resources. Given the rise of food insecurity among college students during the past decade and post the recent pandemic [1–3, 26], the current study has important public health implications and yields critical insights about the utility of on-campus safety net programs for college students.

This systematic review identified that certain historically marginalized groups and subpopulations of college students were more likely to use food pantries within their university. These groups included Asian, Hispanic, Filipino/Pacific Islander, recipients of student loans, first-generation to college, and international students. These results are in line with emerging data suggesting that Asian, Hispanic, and Black individuals were over twice as likely as their White counterparts to experience food insecurity [27]. In addition, the international student population has received little attention regarding food insecurity and their access to food pantries [12]. A recent review published in 2021 reported that international students were more likely to be at risk for food insecurity than domestic students and faced unique challenges due to housing and financial issues [28]. International students in one cross-sectional study identified factors such as the high cost of rent, high tuition fees, shortage of affordable housing, lack of student loans and working opportunities, and lack of information that affected their ability to study and live abroad [29]. Many of these factors have been associated with food insecurity [30, 31].

The current study elicited numerous barriers and challenges that students face when accessing food pantries within their educational institutions. While some studies reported challenges such as unclear eligibility criteria, poor quality of food or expired items, inconvenient hours of operation, and insufficient culturally appropriate foods, nearly all studies in this systematic review found that stigma was a barrier to using a food pantry [12, 19, 21–24]. While some studies found that participants had internalized stigma associated with using food pantries, others reported structural stigma through messages promoted by their institutions and feelings of embarrassment they received from other students watching them carry food pantry bags [12, 19, 21–24]. These findings demonstrate that barriers to food access can be systemic, economic, logistical, social, and psychological [22].

Although this study was the first systematic review to examine the predictors, barriers to, and facilitators of using food pantries, this research is not without limitations. First, these findings may not be generalizable to college students in other countries since all studies were conducted in the US. The included samples also predominantly represented female and undergraduate students. Given that five out of eight studies did not use a random sample of participants [12, 19, 20, 24, 25], this review’s findings might not be applicable to other populations. Findings are also not generalizable to students from community colleges and private universities because all the studies recruited their participants from state universities. In addition, the cross-sectional nature of most studies included in this systematic review does not allow causal relationships to be drawn between student characteristics, barriers to, and facilitators of accessing a food pantry on campus. All studies relied on self-report data, which might have led to social desirability bias. It is also likely that more students accessed the food pantries at the institutions where the studies were conducted. However, due to factors such as stigma, these students might not have participated and were unwilling to disclose the challenges that they faced [21].

## Implications for research and practice

Although campus food pantries are critical safety net programs that alleviate hunger among university students [10], they do not address the root causes of food and nutrition security and less is known about their effectiveness in meeting the nutritional needs of students [32]. Further research is needed to evaluate the effectiveness of university food pantries on improving nutritional outcomes among students and identify policy, systems, and environmental-level strategies to ensure access to nutritious foods among students. Given that food pantries are a crucial form of emergency assistance to college students, there is a strong need for additional research to identify ways through which food pantries and higher educational institutions, as a whole, can better address food insecurity [10].

More research is needed to identify effective interventions that would minimize the stigma and embarrassment associated with the use of campus food pantries, spread awareness about campus food pantries (including eligibility requirements and information on how food pantries work), and normalize the use of food pantries on university campuses (e.g., convenient, central campus location). A previous study found that food pantry programs that partnered with other community resources such as a public library were successful in reducing stigma associated with providing free meals [33]. Hence, identifying strategies to integrate meal programs at public libraries or university libraries while also providing information on important food and social resources in subsequent studies could be worthwhile. Additionally, future research studies should examine the disparities in food access and the unique challenges that students of ethnic minorities, first-generation students, those on student loans, and international students face. For example, certain groups such as international students, might not qualify for federal aid. Hence, assessing alternative strategies and programs that could be useful for these student subgroups is critical. For instance, future studies could design and assess the effectiveness of partnerships between universities and local grocery stores, farms, and ethnic restaurants or stores to provide low-cost nutritious and local foods to students.

From a practical standpoint, addressing food insecurity and stigma will require a nuanced, integrated collaborative approach across disciplines (e.g., public health, dietetics, psychology, agriculture) and institutional departments (e.g., student services, counseling center, health centers [34]. As higher education institutions work towards recruiting and retaining historically marginalized groups of students and international individuals, dedicated staff members and basic needs coordinators who are culturally competent and who can provide a safe and destigmatizing environment will be a priority. Universities need to create and include destigmatizing marketing messages about services like food pantries during student events and fairs, on their websites, social media pages, and course syllabi. Universities can also send monthly reminders regarding food pantries’ location and their hours of operation, how to access the pantries in a confidential manner, and the types of foods that students can access at pantries. There is a need for higher education institutions to identify ways to alleviate the financial burden of higher education for students, such as providing open education resources in addition to developing free cooking and food management classes [22], and provide funds every semester to food pantry administrators so that the food pantries can be stocked with adequate food supplies. Food pantry hours can also be extended during certain times of the semester such as the start, exam periods, and holiday periods when students might need them the most. In addition, leaders of the food pantries and university administrators can partner with local farms and food banks, grocery stores, local restaurants that serve different types of ethnic foods, community gardens, and faith-based organizations to improve access to a variety of fresh produce and culturally acceptable foods. It is also critical for policymakers to revise existing policies related to federal food and nutrition assistance programs and expand eligibility criteria for college students given the changing demographics of this target group in the US.

## Conclusions

This first systematic review provides information about factors that help or pose a challenge to students when using college food pantries and which student subgroups are likely to use an on-campus food pantry. This review showed that participants reported barriers such as stigma, discomfort, embarrassment, and lack of information about a campus food pantry. These challenges need to be systematically addressed with multi-level interventions that span the individual-level, to reduce feelings of discomfort, all the way through structural change at the campus-level to provide greater administrative support to facilitate food pantry operations (e.g., extended hours to meet students’ schedules). Given the academic and health-related impact of food insecurity on students’ overall well-being, campus pantry leaders, university administrators, and policymakers need to prioritize initiatives that effectively improve access to safe, nutritious, and culturally acceptable foods among students.

## Data Availability

The datasets used and/or analysed during the current study available from the corresponding author on reasonable request.

## Abbreviation

US: United States
